# The shallow water equation and the vorticity equation for a change in height of the topography

**DOI:** 10.1371/journal.pone.0178184

**Published:** 2017-06-07

**Authors:** ChaoJiu Da, BingLu Shen, PengCheng Yan, DeShan Ma, Jian Song

**Affiliations:** 1 School of Mathematics and Computer Science Institute, Northwest University for Nationalities, Lanzhou, Gansu, China; 2 College of Atmospheric Sciences, Lanzhou University, Lanzhou, Gansu, China; 3 College of Atmospheric Sciences, Chengdu University of Information Technology, Chengdu, Sichuan, China; 4 Institute of Arid Meteorology of CMA, Lanzhou, Gansu, China; 5 College of Science, Inner Mongolia University of Technology, Hohhot, Inner Mongolia, China; Shanxi University, CHINA

## Abstract

We consider the shallow water equation and the vorticity equations for a variable height of topography. On the assumptions that the atmosphere is incompressible and a constant density, we simplify the coupled dynamic equations. The change in topographic height is handled as the sum of the inherent and changing topography using the perturbation method, together with appropriate boundary conditions of the atmosphere, to obtain the relationship between the relative height of the flow, the inherent topography and the changing topography. We generalize the conservation of the function of relative position, and quantify the relationship between the height of the topography and the relative position of a fluid element. If the height of the topography increases (decreases), the relative position of a fluid element descends (ascends). On this basis, we also study the relationship between the vorticity and the topography to find the vorticity decreasing (increasing) for an increasing (decreasing) height of the topography.

## Introduction

Atmospheric motion is governed by laws of physics described by partial differential equations, which control changes of motion and the thermal state of the atmosphere. Initial and boundary conditions are required to solve these partial differential equations. Initial conditions are defined based on the initial flow distribution, while the boundary conditions are distinguished by the inner and outer boundary conditions. For the motion of the atmosphere, only the lower boundary conditions are required, which is taken as the surface of the topography. As a result of natural variability or human factors, the height of the topography may change, which can alter the variation in atmospheric circulation, the local climate, or even the global climate. The change of the height of the topography may reflect the effect of human activities on the atmospheric motion on a global scale. We focus here on the change in height of the topography, and its effect on the generalized dynamic processes of the atmosphere.

Liao et al. studied the influence of the topography on rainfall, concluding that the change in the height of the topography not only has an effect on the size and distribution of the horizontal and vertical flow fields of the atmosphere, but also alters atmospheric microphysical process, which affects the local climate [1]. The change in height of the topography can also lead to the occurrence of disastrous weather locally [2]. Researchers have long realized that the dynamic uplift of the topography on warm and humid air causes heavy rain [3,4]. For example, the change in the height of the topography is inversely correlated with the precipitation intensity of a ridge [5]. Because atmospheric flow is a highly nonlinear system, the small-scale variation of the height of the topography may lead to nonlinear instability, Duan et al. did a systematic study on the nonlinear instability of atmospheric motion [6,7]. Nonlinearity is the source of the chaotic behavior of the atmosphere, which mathematical theory attributes to instabilities in the solutions to the governing differential equations, leading to difficulties in numerical weather prediction, for the nonlinearity, Sun et al. did the mathematics and physics fundamental research, and gained a series of conclusion [8–13], the research result can partly explain nonlinear effect of the differential equations. Feng GuoLin and Huang JianPing et al. used historical observational data to improve the results of numerical simulations, and possible physical mechanism of water vapor transport [14–19]. The change in the topography also impacts the forecasts of numerical models [20]. For a slowly changing height of the topography, Da ChaoJiu et al. studied the shallow water equations when the local horizontal divergence changes slowly, modifying the equilibrium between the local horizontal divergence and the local change in the thickness of the atmosphere [21–23].

The influence of the topography on the motion of atmosphere is mainly thermal and dynamic effect, the former is mainly the action on the temperature and humidity of the atmosphere, the latter is mainly the action on the velocity and pressure. In this paper, we only do the research on the change of the topography height.

## The shallow water equation for a change of the topography

**Fig 1:**
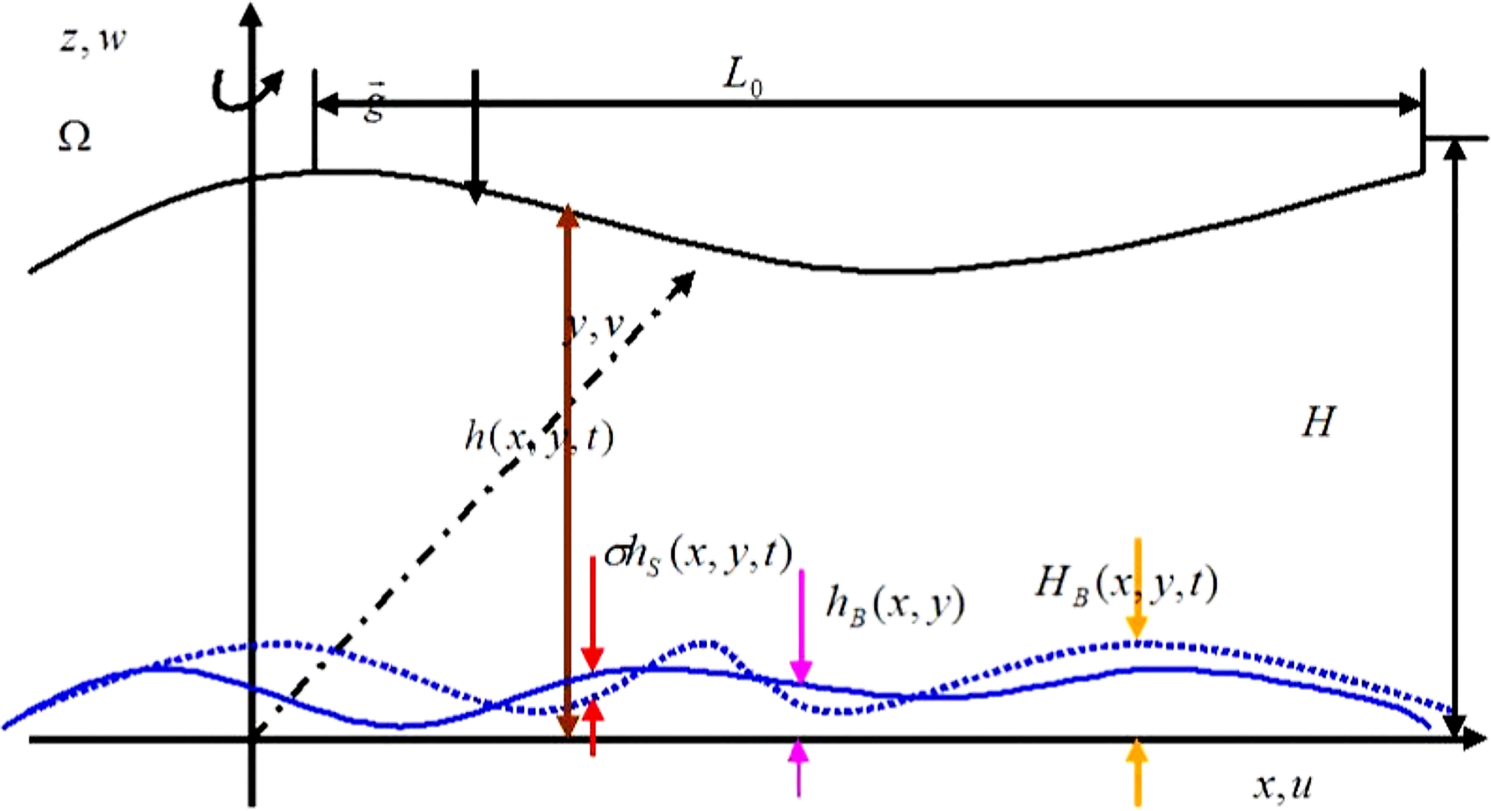
Conceptual diagram showing the change in height of the topography.

For the barotropic flow, the simplest model is the shallow water model. We assume that the whole atmosphere is one layer of constant density, incompressible, satisfies the static approximation, together with the *β*-plane approximation. Fig 1 gives the conceptual diagram showing the change in the height of the topography. For a single layer, uniform-density fluid (*ρ* = constant) above the plane *z* = 0, the height of the fluid surface is *h*(*x*,*y*,*t*). In view of the effect of the earth on the atmosphere and ocean, the force of the potential function Φ can be thought of as the vector $\vec{g}$ perpendicular to the plane *z* = 0. Here, the rotation of the Earth $\vec{\Omega }=k\Omega$ is parallel to the *z*-coordinate axis. The topographical boundary has two parts, one being the inherent underlying surface of the atmosphere or the ocean *h*_*B*_(*x*,*y*,*t*), the other being the part that slowly changes in time *h*_*S*_(*x*,*y*,*t*), written as

$$z\equiv H_{B}(x,y,t)=h_{B}(x,y)+\sigma h_{S}(x,y,t).$$ (1)

Here, the parameter *σ* takes a small value, which represents the gradual change in topography. This is based on the requirement of the perturbation method, and also meet the slowly change character of the topography, and can describe the real motion of the atmosphere. The velocity components parallel to the coordinate axes *x*, *y* and *z* are *u*, *v* and *w*, respectively, assuming furthermore the fluid is inviscid [24,25].

From the assumptions of incompressibility and a constant density, the continuity equation can be simplified to

$$\frac{\partial u}{\partial x}+\frac{\partial v}{\partial y}+\frac{\partial w}{\partial z}=0$$ (2A)

and the horizontal momentum equations are

$$\frac{\partial u}{\partial t}+u\frac{\partial u}{\partial x}+v\frac{\partial u}{\partial y}-fv=-g\frac{\partial h}{\partial x}$$ (2B)

and

$$\frac{\partial v}{\partial t}+u\frac{\partial v}{\partial x}+v\frac{\partial v}{\partial y}+fu=-g\frac{\partial h}{\partial y}.$$ (2C)

Here, define the depth of fluid as [24,25]

$$H=h-H_{B}(x,y,t)=h-h_{B}(x,y)-\sigma h_{S}(x,y,t).$$ (3)

Integrating the continuity Eq (2A) with respect to *z* above the interval [*H*_*B*_(*x*,*y*,*t*),*h*], while noting the normal velocity on the rigid surface *z* = *H*_*B*_(*x*,*y*,*t*) is zero [26], and the kinematic and the dynamic conditions on the free surface *z* = *h*(*x*,*y*,*t*), we obtain

$$\frac{\partial H}{\partial t}+\frac{\partial }{\partial x}(uH)+\frac{\partial }{\partial y}(vH)=0,$$ (4)

which is consistent with the shallow water equations presented formally in reference [25]. The difference is that the function *H* in reference [25] lacks the temporal variation term *σh*_*S*_(*x*,*y*,*t*) appearing in Eq (4) for consideration of the evolution of the height of the topography. Eqs (2) and (4) are our modified shallow water equations. The unknowns *u*, *v* and *h* are associated with the parameters defining *σ*, which is relatively small. Supposing that the variable *u* is the sum of two terms

$$u(x,y,t,\sigma )=u_{0}(x,y,t)+\sigma u_{1}(x,y,t),$$ (5A)

then the first term *u*_0_(*x*,*y*,*t*) is independent of the change of the topography, while the second term *u*_1_(*x*,*y*,*t*) is associated with the change of the topography. Using the parameter *σ* to describe the influence of the topography, the variables *v*, *w* and *h* can similarly be written as

$$v(x,y,t,\sigma )=v_{0}(x,y,t)+\sigma v_{1}(x,y,t),$$ (5B)

$$w(x,y,t,\sigma )=w_{0}(x,y,t)+\sigma w_{1}(x,y,t)+\sigma^{2}w_{2}(x,y,t),$$ (5C)

and

$$h(x,y,t,\sigma )=h_{0}(x,y,t)+\sigma h_{1}(x,y,t).$$ (5D)

As the horizontal velocity is bigger than the vertical velocity for two or three orders of magnitude for the motion of the atmosphere, the horizontal velocity expand to order *σ*, while the vertical velocity to *σ*^2^, this will help to ensure high accuracy. Inserting Eq (5) into the shallow water Eqs (2) and (4), and expanding to obtain terms of *O*(*σ*^0^), gives

$$\frac{\partial u_{0}}{\partial t}+u_{0}\frac{\partial u_{0}}{\partial x}+v_{0}\frac{\partial u_{0}}{\partial y}-fv_{0}=-g\frac{\partial h_{0}}{\partial x},$$ (6A)

$$\frac{\partial v_{0}}{\partial t}+u_{0}\frac{\partial v_{0}}{\partial x}+v_{0}\frac{\partial v_{0}}{\partial y}+fu_{0}=-g\frac{\partial h_{0}}{\partial x}$$ (6B)

and

$$\frac{\partial (h_{0}-h_{B})}{\partial t}+u_{0}\frac{\partial (h_{0}-h_{B})}{\partial x}+v_{0}\frac{\partial (h_{0}-h_{B})}{\partial y}+(h_{0}-h_{B})(\frac{\partial u_{0}}{\partial x}+\frac{\partial v_{0}}{\partial y})=0.$$ (6C)

Invoking a zero normal-velocity component on the rigid base to obtain the vertical velocity *w*(*x*,*y*,*t*,*σ*), where *w*_0_ is the term of *O*(*σ*^0^), gives

$$w_{0}=(h_{B}-z)(\frac{\partial u_{0}}{\partial x}+\frac{\partial v_{0}}{\partial y})+u_{0}\frac{\partial h_{B}}{\partial x}+v_{0}\frac{\partial h_{B}}{\partial y}.$$ (6D)

Using Eq (6C) to eliminate $\frac{\partial u_{0}}{\partial x}+\frac{\partial v_{0}}{\partial y}$ in Eq (6D) results in

$$w_{0}=(h_{B}-z)\frac{1}{\left(h_{B}-h_{0}\right)}\frac{d_{0}(h_{0}-h_{B})}{dt}+u_{0}\frac{\partial h_{B}}{\partial x}+v_{0}\frac{\partial h_{B}}{\partial y}.$$ (7)

In this way, the function *w*_0_ can be written as a function of the topography, where the term $\frac{d_{0}(h_{0}-h_{B})}{dt}$ has the form

$$\frac{d_{0}(h_{0}-h_{B})}{dt}=\frac{\partial (h_{0}-h_{B})}{\partial t}+u_{0}\frac{\partial (h_{0}-h_{B})}{\partial x}+v_{0}\frac{\partial (h_{0}-h_{B})}{\partial y},$$ (8)

which can also be interpreted as the zero-order expansion of the full derivative.

The *O*(*σ*^1^) equations are

$$\frac{\partial u_{1}}{\partial t}+\frac{\partial u_{0}u_{1}}{\partial x}+v_{0}\frac{\partial u_{1}}{\partial y}+v_{1}\frac{\partial u_{0}}{\partial y}-fv_{1}=-g\frac{\partial h_{1}}{\partial x},$$ (9A)

$$\frac{\partial v_{1}}{\partial t}+u_{0}\frac{\partial v_{1}}{\partial x}+u_{1}\frac{\partial v_{0}}{\partial x}+\frac{\partial v_{0}v_{1}}{\partial y}+fu_{1}=-g\frac{\partial h_{1}}{\partial y},$$ (9B)

$$\frac{\partial (h_{1}-h_{S})}{\partial t}+\frac{\partial u_{0}(h_{1}-h_{S})}{\partial x}+\frac{\partial u_{1}(h_{0}-h_{B})}{\partial x}+\frac{\partial v_{0}(h_{1}-h_{S})}{\partial y}+\frac{\partial v_{1}(h_{0}-h_{B})}{\partial y}=0,$$ (9C)

and

$$w_{1}=(h_{B}-z)(\frac{\partial u_{1}}{\partial x}+\frac{\partial v_{1}}{\partial y})+h_{S}(\frac{\partial u_{0}}{\partial x}+\frac{\partial v_{0}}{\partial y})+u_{0}\frac{\partial h_{S}}{\partial x}+u_{1}\frac{\partial h_{B}}{\partial x}+v_{0}\frac{\partial h_{S}}{\partial y}+v_{1}\frac{\partial h_{B}}{\partial y}.$$ (9D)

From Eq (6C), we have

$$(\frac{\partial u_{0}}{\partial x}+\frac{\partial v_{0}}{\partial y})=\frac{1}{\left(h_{B}-h_{0}\right)}\frac{d_{0}(h_{0}-h_{B})}{dt}$$ (10)

which is substituted into Eq (9C) to give

$$(h_{0}-h_{B})(\frac{\partial u_{1}}{\partial x}+\frac{\partial v_{1}}{\partial y})=-\frac{d_{0}(h_{1}-h_{S})}{dt}-\frac{\left(h_{1}-h_{S}\right)}{\left(h_{B}-h_{0}\right)}\frac{d_{0}(h_{0}-h_{B})}{dt}+\frac{\partial (h_{0}-h_{B})}{\partial t}-\frac{d_{1}(h_{0}-h_{B})}{dt},$$ (11)

where

$$\frac{d_{1}(h_{0}-h_{B})}{dt}=\frac{\partial (h_{0}-h_{B})}{\partial t}+u_{1}\frac{\partial (h_{0}-h_{B})}{\partial x}+v_{1}\frac{\partial (h_{0}-h_{B})}{\partial y}.$$ (12)

This equation can also be interpreted as the first-order expansion of the full derivative, where $\frac{d_{0}(h_{1}-h_{S})}{dt}$ is defined similarly to Eq (8), but is not repeated here. Substituting Eq (10) and Eq (11) into Eq (9D) gives

$$\begin{array}{l} w_{1}=\frac{h_{B}-z}{h_{0}-h_{B}}[\frac{\partial (h_{0}-h_{B})}{\partial t}-\frac{d_{0}(h_{1}-h_{S})}{dt}-\frac{\left(h_{1}-h_{S}\right)}{\left(h_{B}-h_{0}\right)}\frac{d_{0}(h_{0}-h_{B})}{dt}-\frac{d_{1}(h_{0}-h_{B})}{dt}]\\ +\frac{h_{S}}{h_{B}-h_{0}}\frac{d_{0}(h_{0}-h_{B})}{dt}+u_{0}\frac{\partial h_{S}}{\partial x}+u_{1}\frac{\partial h_{B}}{\partial x}+v_{0}\frac{\partial h_{S}}{\partial y}+v_{1}\frac{\partial h_{B}}{\partial y} \end{array},$$ (13)

which is *w*_1_ expressed in terms of the topography, similarly to Eq (7).

The *O*(*σ*^2^) equations are

$$u_{1}\frac{\partial u_{1}}{\partial x}+v_{1}\frac{\partial u_{1}}{\partial y}=0,$$ (14A)

$$u_{1}\frac{\partial v_{1}}{\partial x}+v_{1}\frac{\partial v_{1}}{\partial y}=0,$$ (14B)

$$\frac{\partial u_{1}(h_{1}-h_{S})}{\partial x}+\frac{\partial v_{1}(h_{1}-h_{S})}{\partial y}=0,$$ (14C)

and

$$w_{2}=h_{S}(\frac{\partial u_{1}}{\partial x}+\frac{\partial v_{1}}{\partial y})+u_{1}\frac{\partial h_{S}}{\partial x}+v_{1}\frac{\partial h_{S}}{\partial y}.$$ (14D)

Substituting Eq (11) into Eq (14D) gives

$$\begin{array}{l} w_{2}=\frac{h_{S}}{h_{0}-h_{B}}[\frac{\partial (h_{0}-h_{B})}{\partial t}-\frac{d_{0}(h_{1}-h_{S})}{dt}-\frac{\left(h_{1}-h_{S}\right)}{\left(h_{B}-h_{0}\right)}\frac{d_{0}(h_{0}-h_{B})}{dt}-\frac{d_{1}(h_{0}-h_{B})}{dt}]\\ +u_{1}\frac{\partial h_{S}}{\partial x}+v_{1}\frac{\partial h_{S}}{\partial y} \end{array},$$ (15)

Adding Eq (7) to Eq (13), multiplying by *σ*, and then adding to Eq (15) multiplied by *σ*^2^ gives

$$\begin{array}{l} \frac{dz}{dt}\equiv w=\frac{h_{B}-z}{h_{B}-h_{0}}\frac{d_{0}(h_{0}-h_{B})}{dt}+u_{0}\frac{\partial h_{B}}{\partial x}+v_{0}\frac{\partial h_{B}}{\partial y}\\ +\sigma \frac{h_{B}-z}{h_{0}-h_{B}}[\frac{\partial (h_{0}-h_{B})}{\partial t}-\frac{d_{0}(h_{1}-h_{S})}{dt}-\frac{\left(h_{1}-h_{S}\right)}{\left(h_{B}-h_{0}\right)}\frac{d_{0}(h_{0}-h_{B})}{dt}-\frac{d_{1}(h_{0}-h_{B})}{dt}]\\ +\frac{\sigma h_{S}}{h_{B}-h_{0}}\frac{d_{0}(h_{0}-h_{B})}{dt}+\sigma u_{0}\frac{\partial h_{S}}{\partial x}+\sigma u_{1}\frac{\partial h_{B}}{\partial x}+\sigma v_{0}\frac{\partial h_{S}}{\partial y}+\sigma v_{1}\frac{\partial h_{B}}{\partial y}\\ +\frac{\sigma^{2}h_{S}}{h_{0}-h_{B}}[\frac{\partial (h_{0}-h_{B})}{\partial t}-\frac{d_{0}(h_{1}-h_{S})}{dt}-\frac{\left(h_{1}-h_{S}\right)}{\left(h_{B}-h_{0}\right)}\frac{d_{0}(h_{0}-h_{B})}{dt}-\frac{d_{1}(h_{0}-h_{B})}{dt}]\\ +\sigma^{2}u_{1}\frac{\partial h_{S}}{\partial x}+\sigma^{2}v_{1}\frac{\partial h_{S}}{\partial y} \end{array},$$ (16)

Hence, the relationship between the vertical position *z*, the topography and the horizontal velocity is now given. If the topography is constant, that is to say *σ* = 0, Eq (16) can be written as

$$\frac{d}{dt}\ln\frac{z-h_{B}}{h-h_{B}}=0$$ (17)

or

$$\frac{d}{dt}(\frac{z-h_{B}}{h-h_{B}})=0,$$ (18)

which illustrates that the relative-position function $\frac{z-h_{B}}{h-h_{B}}$ regulated the motion of each fluid element conserves in the shallow water, which is a well-known condition. The function $\frac{z-h_{B}}{h-h_{B}}$ is the relative height of the fluid element from the bottom boundary, clearly being zero on the bottom boundary (for *z* = *h*_*B*_) and one on the free surface (for *z* = *h*). Meanwhile, the horizontal velocity is independent of *z*, meaning that the flow moves parallel to *z*. Moreover, the relative position of a fluid element within a column remains unchanged for a lengthening or shortening column [24,25]. If the height of the topography changes with time, Eq (16) gives the relationship between the position *z* of the fluid element, the topography and the velocity, and is complex and highly nonlinear. The first line on the right-hand side of Eq (16) is the zero-order approximation, which lacks the changing of the topography, with only the inherent topography being present. The second and third lines on the right-hand side of Eq (16) correspond to the first-order approximation, where a change in the topography is accounted for through *h*_*S*_, including the nonlinear interaction between *h*_*S*_ and *h*_*B*_. The fourth and fifth lines on the right-hand side of Eq (16) represent the second-order approximation, which contains the topography term *h*_*S*_ and nonlinear interaction between *h*_*S*_ and *h*_*B*_. In general, the change of the topography is small, because the parameter characterizing the degree of the change of the topography *σ* is small. For these reasons, Eq (16) can be simplified by omitting the second-order terms. As *σh*_*S*_ in $\frac{\sigma h_{S}}{\left(h_{B}-h_{0}\right)}\frac{d_{0}(h_{0}-h_{B})}{dt}$ is also small, this term may also be omitted. As the term $\frac{\partial h_{S}}{\partial x}$ is the rate of change of slowly varying topography *h*_*S*_ with respect to the longitude x, can also be omitted; and the term $\frac{\partial h_{S}}{\partial y}$ is the rate of change of *h*_*S*_ with respect to the latitude y, can also be omitted. Hence, Eq (16) may be simplified to

$$\begin{array}{l} \frac{dz}{dt}=\frac{h_{B}-z}{h_{B}-h_{0}}\frac{d_{0}(h_{0}-h_{B})}{dt}+u_{0}\frac{\partial h_{B}}{\partial x}+v_{0}\frac{\partial h_{B}}{\partial y}\\ +\sigma \frac{h_{B}-z}{h_{0}-h_{B}}[\frac{\partial (h_{0}-h_{B})}{\partial t}-\frac{d_{0}(h_{1}-h_{S})}{dt}-\frac{\left(h_{1}-h_{S}\right)}{\left(h_{0}-h_{B}\right)}\frac{d_{0}(h_{0}-h_{B})}{dt}-\frac{d_{1}(h_{0}-h_{B})}{dt}]\\ +\sigma u_{1}\frac{\partial h_{B}}{\partial x}+\sigma v_{1}\frac{\partial h_{B}}{\partial y} \end{array},$$ (19)

by which (5A) and (5B) give

$$u_{0}\frac{\partial h_{B}}{\partial x}+v_{0}\frac{\partial h_{B}}{\partial y}+\sigma u_{1}\frac{\partial h_{B}}{\partial x}+\sigma v_{1}\frac{\partial h_{B}}{\partial y}=u\frac{\partial h_{B}}{\partial x}+v\frac{\partial h_{B}}{\partial y}$$ (20)

for simplifying Eq (19) to

$$\frac{1}{z-h_{B}}\frac{d(z-h_{B})}{dt}-\frac{1}{h_{0}-h_{B}}\frac{d(h_{0}-h_{B})}{dt}=\sigma \frac{1}{h_{0}-h_{B}}[\frac{d_{0}(h_{1}-h_{S})}{dt}+\frac{\left(h_{1}-h_{S}\right)}{\left(h_{B}-h_{0}\right)}\frac{d_{0}(h_{0}-h_{B})}{dt}]=0.$$ (21)

or

$$\begin{array}{l} \frac{1}{z-h_{B}}\frac{d(z-h_{B})}{dt}-\frac{1}{h_{0}-h_{B}}\frac{d(h_{0}-h_{B})}{dt}\\ =\sigma \frac{h_{1}-h_{S}}{h_{0}-h_{B}}[\frac{1}{h_{1}-h_{S}}\frac{d_{0}(h_{1}-h_{S})}{dt}-\frac{1}{h_{0}-h_{B}}\frac{d_{0}(h_{0}-h_{B})}{dt}] \end{array},$$ (22A)

so that Eq (22A) may be written as

$$\frac{d}{dt}\ln\frac{z-h_{B}}{h_{0}-h_{B}}=\sigma \frac{h_{1}-h_{S}}{h_{0}-h_{B}}\frac{d_{0}}{dt}\ln\frac{h_{1}-h_{S}}{h_{0}-h_{B}},$$ (22B)

where the differential operator $\frac{d_{0}}{dt}$ in Eq (22) is defined after Eq (8). In comparing Eq (22) with Eq (18), we see that the relative-position function $\frac{z-h_{B}}{h-h_{B}}$ is not conserved. The change of the topography through the relative position function $\frac{z-h_{B}}{h-h_{B}}$ illustrates that if the topography *h*_*S*_ changes with the time, this function will adjust to adapt to the change of the topography. Therefore, Eq (22) indicates the motion of the atmosphere more accurately than (18). To help understand Eq (22), we study one special case by supposing *u*_0_, *v*_0_ and *h*_0_ in Eq (6) have the solutions

$$u_{0}=0,\;v_{0}=0,\;h_{0}=D,$$ (23)

where *D* is constant, and Eq (23) is a specific solution of Eq (6). This means the atmosphere in the horizontal direction is stationary, and the topography is quasi horizontal, the case is simple, but has the characteristics of the motion of the atmosphere. With this assumption, Eq (9) can be simplified to

$$\frac{\partial u_{1}}{\partial t}-fv_{1}=-g\frac{\partial h_{1}}{\partial x},$$ (24A)

$$\frac{\partial v_{1}}{\partial t}+fu_{1}=-g\frac{\partial h_{1}}{\partial y},$$ (24B)

$$\frac{\partial (h_{1}-h_{S})}{\partial t}+\frac{\partial u_{1}(D-h_{B})}{\partial x}+\frac{\partial v_{1}(D-h_{B})}{\partial y}=0,$$ (24C)

and

$$w_{1}=(h_{B}-z)(\frac{\partial u_{1}}{\partial x}+\frac{\partial v_{1}}{\partial y})+u_{1}\frac{\partial h_{B}}{\partial x}+v_{1}\frac{\partial h_{B}}{\partial y},$$ (24D)

Eq (22B) also simplified to

$$(D-h_{B})\frac{d}{dt}\ln\frac{z-h_{B}}{D-h_{B}}=\sigma (h_{1}-h_{S})\frac{\partial }{\partial t}\ln\frac{h_{1}-h_{S}}{D-h_{B}}.$$ (25)

As the inherent topography *h*_*B*_ is time-independent, Eq (25) has the new form

$$(D-h_{B})\frac{d}{dt}\ln\frac{z-h_{B}}{D-h_{B}}+\sigma \frac{\partial (h_{S}-h_{1})}{\partial t}=0,$$ (26)

where *D*−*h*_*B*_ > 0. If $\sigma \frac{\partial (h_{S}-h_{1})}{\partial t}>0$, this indicates *h*_*S*_ increases in time, meaning that the height of the topography increases. Thus, the term $\frac{d}{dt}\ln\frac{z-h_{B}}{D-h_{B}}$ is negative, implying that for the stretching or contracting of a fluid element, the relative position of the fluid element in the fluid column descends to balance the increasing height of the topography. Conversely, if $\sigma \frac{\partial (h_{S}-h_{1})}{\partial t}<0$, and *h*_*S*_ decreases with time, the height of the topography decreases for positive $\frac{d}{dt}\ln\frac{z-h_{B}}{D-h_{B}}$. Hence, the relative position of a fluid element within a fluid column must ascend to balance a decline in the height of the topography.

## The vorticity equation for a change of the topography

The components of vorticity are written as

$$\varpi_{x}=\frac{\partial w}{\partial y}-\frac{\partial v}{\partial z},$$ (27A)

$$\varpi_{y}=\frac{\partial u}{\partial z}-\frac{\partial w}{\partial x},$$ (27B)

and

$$\varpi_{z}=\frac{\partial v}{\partial x}-\frac{\partial u}{\partial y}.$$ (27C)

For the shallow water model, *u* and *v* are *z*-independent, while a scale analysis tells us that the horizontal component of relative vorticity is smaller than the vertical component. To study the vertical component, we write

$$\zeta \equiv \varpi_{z}=\zeta_{0}+\sigma \zeta_{1}$$ (28)

and only consider the horizontal momentum Eq (2) to obtain

$$\frac{d\zeta }{dt}+(f+\zeta )(\frac{\partial u}{\partial x}+\frac{\partial v}{\partial y})=0$$ (29)

which defines the behavior of the relative vorticity with respect to the divergence of the absolute vortex filament [24, 25]. The deformation of Eq (4) is

$$\frac{dH}{dt}+H(\frac{\partial u}{\partial x}+\frac{\partial v}{\partial y})=0,$$ (30)

which, with the help of Eqs (29) and (30), we eliminate $\frac{\partial u}{\partial x}+\frac{\partial v}{\partial y}$ to obtain

$$H\frac{d\zeta }{dt}=(f+\zeta )\frac{dH}{dt}.$$ (31)

Eqs (31) is the conservative laws on vorticity, this indicates the vorticity is conserved in the case of the changing topography. Finally, inserting Eqs (3) and (28) into Eq (31) gives

$$\frac{d}{dt}(\frac{h-h_{B}-\sigma h_{S}}{f+\zeta })=0.$$ (32)

Hence, if the height of the topography increases (decreases), meaning that *h*_*S*_ increases (decreases) in time, the relative vorticity *ζ* must decrease (increase).

## Conclusions

Under the influence of global warming and other damaging human activities, the height of the topography may change, which will have an impact on the motion of the atmosphere. A method for quantitatively describing this impact is given here. In targeting the change of the topography in time with the help of the perturbation method, together with the pertinent boundary conditions, we quantitatively study the changing of the relative-position function $\frac{z-h_{B}}{h-h_{B}}$ and the relative vorticity *ζ* for a changing topography. We obtain the following conclusions: 1. For a changing topography, the relative-position function is not conserved and must therefore adjust to the topography; 2. In cases for which the height of the topography increases, the relative position of a fluid element in a fluid column must descend; conversely, if the height of the topography decreases, the relative position of a fluid element in a fluid column must ascend; 3. If the height of the topography increases (decreases), the vorticity decreases (increases).
